# Association between Maternal Exposure to di(2-ethylhexyl) Phthalate and Reproductive Hormone Levels in Fetal Blood: The Hokkaido Study on Environment and Children's Health

**DOI:** 10.1371/journal.pone.0109039

**Published:** 2014-10-08

**Authors:** Atsuko Araki, Takahiko Mitsui, Chihiro Miyashita, Tamie Nakajima, Hisao Naito, Sachiko Ito, Seiko Sasaki, Kazutoshi Cho, Tamiko Ikeno, Katsuya Nonomura, Reiko Kishi

**Affiliations:** 1 Center for Environmental and Health Sciences, Hokkaido University, Sapporo, Hokkaido, Japan; 2 Department of Urology, Hokkaido University Hospital, Sapporo, Hokkaido, Japan; 3 Graduate School of Medicine, Nagoya University, Nagoya, Aichi, Japan; 4 Department of Public Health, Graduate School of Medicine, Hokkaido University, Sapporo, Hokkaido, Japan; 5 Department of Obstetrics and Genecology, Hokkaido University Hospital, Sapporo, Hokkaido, Japan; 6 Department of Renal and Genitourinary Surgery, Graduate School of Medicine, Hokkaido University, Sapporo, Hokkaido, Japan; University of Kansas Medical Center, United States of America

## Abstract

Prenatal di(2-ethylhexyl) phthalate (DEHP) exposure can produce reproductive toxicity in animal models. Only limited data exist from human studies on maternal DEHP exposure and its effects on infants. We aimed to examine the associations between DEHP exposure *in utero* and reproductive hormone levels in cord blood. Between 2002 and 2005, 514 pregnant women agreed to participate in the Hokkaido Study Sapporo Cohort. Maternal blood samples were taken from 23–35 weeks of gestation and the concentration of the primary metabolite of DEHP, mono(2-ethylhexyl) phthalate (MEHP), was measured. Concentrations of infant reproductive hormones including estradiol (E2), total testosterone (T), and progesterone (P4), inhibin B, insulin-like factor 3 (INSL3), steroid hormone binding globulin, follicle-stimulating hormone, and luteinizing hormone were measured from cord blood. Two hundred and two samples with both MEHP and hormones' data were included in statistical analysis. The participants completed a self-administered questionnaire regarding information on maternal characteristics. Gestational age, birth weight and infant sex were obtained from birth records. In an adjusted linear regression analysis fit to all study participants, maternal MEHP levels were found to be associated with reduced levels of T/E2, P4, and inhibin B. For the stratified analyses for sex, inverse associations between maternal MEHP levels T/E2, P4, inhibin B, and INSL3 were statistically significant for males only. In addition, the MEHP quartile model showed a significant p-value trend for P4, inhibin B, and INSL3 decrease in males. Since inhibin B and INSL3 are major secretory products of Sertoli and Leydig cell, respectively, the results of this study suggest that DEHP exposure *in utero* may have adverse effects on both Sertoli and Leydig cell development in males, which agrees with the results obtained from animal studies. Comprehensive studies investigating phthalates' exposure in humans, as well as their long-term effects on reproductive development are needed.

## Introduction

Diesters of phthalic acid (phthalates) have been used as plasticizers for various plastic compounds, such as toys, food containers, furniture, personal care products, medical devices, and housing materials. Phthalates are not chemically bonded to polyvinyl chloride (PVC) in plastic products and, as a result, they can leach and migrate into the air, foodstuffs, and other materials. Consequently, humans are constantly exposed to phthalates and biomonitoring studies have shown the widespread exposure of the general population to these chemicals [1]–[4].

Di(2-ethylhexyl) phthalate (DEHP) is one of the major phthalate compounds and constitutes more than 50% of the phthalates used in production in Japan [5]. Phthalates are known to exert endocrine-disrupting effects, which have been the cause of some concern [6]. Animal studies have shown that fetal exposure to DEHP may induce abnormalities in the reproductive system, reduce testosterone (T) and insulin-like factor 3 (INSL3) levels, and cause disruption to Leydig and Sertoli cell maturation [7]–[10].

In a study that looked at the effects of DEHP on humans, male study participants, who were mainly recruited from an infertility clinic and had elevated levels of phthalate metabolites in their urine, were found to exhibit lower sperm concentrations and T levels, higher levels of follicle-stimulating hormone (FSH), and have a higher incidence of damaged sperm DNA [11]–[16]. In female study participants the phthalate exposure level was associated with physical signs of puberty, such as breast enlargement, the growth of pubic and axillary hair, premature thelarche, and central precocious puberty [17]–[19].

However, epidemiological studies on the effects of DEHP exposure of infants *in utero* or in early life are limited. Swan et al. [20] indicated that maternal phthalate exposure was inversely related to the anogenital distance (AGI) of male infants and Huang et al. [21] reported that the levels of phthalates in amniotic fluid were inversely related to the AGI of female infants. Only two studies have examined the effects of phthalates on the reproductive hormone levels of infants. In one study maternal urinary metabolites were measured and the level of DEHP was found to be inversely correlated with free T (fT) and the fT/estradiol (E2) ratio in cord blood among female infants [22]. In another study reproductive hormone levels were measured from males with cryptorchidism and healthy control subjects at three months of age, as well as phthalate metabolites in breast milk. Phthalate metabolites (dimethyl phthalate (DMP), diethyl phthalate (DEP), and dibutyl phthalate (DBP)) positively correlated with the luteinizing hormone (LH)/fT ratio, and DEP and DBP showed positive correlations with steroid hormone-binding globulin (SHBG), while DBP was also found to inversely correlate with fT [23]. However, phthalate exposure has not been found to relate to cryptorchidism directly [23].

Although there is some evidence to suggest that fetal and neonatal phthalate exposure has an adverse effect on human reproductive development in both male and female infants, comprehensive studies are limited. Therefore, the aim of this study was to examine the associations between DEHP exposure *in utero* and reproductive hormone levels in cord blood in the general population in Japan.

## Materials and Methods

### Population

This prospective birth cohort study was based on the Sapporo Cohort, Hokkaido Study on Environment and Child Health [24], [25]. Study details regarding the population, data collection, sampling of the biological specimens, and the contents of the questionnaire have been described elsewhere [24], [25]. Briefly, native Japanese women living in Sapporo City or the surrounding areas were enrolled into the study at 23–35 weeks of gestation from July 2002 to October 2005 at the Sapporo Toho Hospital, which is an obstetrics and gynecology hospital in Sapporo, Hokkaido, Japan. Among the 1796 pregnant females approached, 25% were excluded as they were enrolled in the Japanese cord blood bank or delivered the baby at another hospital. Ultimately, 514 pregnant females were enrolled in this study (participation rate: 28.6%).

### Assessment of exposure

Blood samples of approximately 40 mL were obtained from participants at the time of their hospital examination after recruitment. If the blood sample could not be taken during pregnancy due to maternal anemia, a blood sample was collected during hospitalization within a week after delivery. All samples were stored at −80°C until analysis. The concentration of mono(2-ehtylhexyl) phthalate (MEHP), which is the primary metabolite of DEHP, in the blood was determined. The method of Instrumental analysis, general method validation, and quality controls were previously described elsewhere with the following modifications of sample preparation in this study [26]. Blood samples (30 uL) were mixed with 120 uL 1N HCl, 350 uL saturated saline solution and 50 uL of 10 uM MEHP-d as an internal standard. MEHP was then extracted twice with 500 ul ethyl acetate after shaking for 15 min. The ethyl acetate layer was evaporated, and the sediments were dissolved into 40 uL ethyl acetate. After adding 20 uL N-methyl-N-(tert-butyldimethylsilyl) trifluoroacetamide (GL Sciences, Tokyo, Japan), the tube was left at room temperature for 60 min, and the MEHP tert-butyldimethylsilyl derivative concentration formed was measured by a GC-MS under the analytical conditions mentioned previously [26]. Under these conditions, the extraction recovery of MEHP was 95.6±1.9 (n = 6, mean±SD) [26]. Two ions, m/z 227 and 339 for quantification ion and confirmation ion, respectively, were used to detect MEHP [27]. The limit of detection (LODs) was 1 pmol/mL (0.278 ng/mL). MEHP levels in a tube containing the same medium as the reaction vial were measured to determine background levels. To exclude the possibility of environmental contamination of DEHP, all glassware used for MEHP measurements was heated at 200°C for 2 h. Ultimately, MEHP level was available in 493 maternal blood samples.

### Outcome measures

At the time of delivery, a blood sample (10–30 mL) was collected from the umbilical cord and stored at −80°C until analysis. Concentrations of E2, total T, and progesterone (P4) were measured using liquid chromatography–tandem mass spectrometry (LC–MS/MS) [28], [29]. An immunoradiometric assay (IRMA) was used to measure the concentrations of LH (Spac-S LH Kit, TFB, Inc., Tokyo Japan), FSH (Spac-S FSH Kit, TFB, Inc., Tokyo Japan), SHBG (IRMA-Count SHBG, Siemens, Berlin, Germany), and prolactin (PRL) (Spac-S Prolactin kit, TFB, Inc., Tokyo, Japan). The concentration of inhibin B was measured by an enzyme-linked immunosorbent assay (ELISA) (inhibin B Gen II ELISA, Beckman Coulter, Inc., CA, USA). The concentration of INSL3 was measured using an enzyme immunoassay (EIA) (insulin-like 3 (INSL3)/RLF (human) EIA kit, Phoenix Pharmaceuticals, Inc., CA, USA). Inhibin B is a marker of Sertoli cell function [30], and INSL3 is a major Leydig cells product and an early marker of the testicular descent during fetal [31], and All reproductive hormone measurements were performed at Aska Pharma Medical Co., Ltd (Kanagawa, Japan).

### Questionnaire and medical record

The participants completed a self-administered questionnaire regarding information on maternal age, educational level, household income, smoking status, alcohol intake, and medical history. Maternal alcohol intake was classified into two categories: no, who never intake alcohol since the first trimester, and yes, who still drink alcohol after the first trimester [25]. Maternal smoking status during pregnancy was classified into two categories: non-smokers, who never smoked or quit smoking during the first trimester, and smokers, who still smoked after the first trimester [25]. Medical records were obtained at delivery for information regarding pre-pregnancy body mass index (BMI), pregnancy complications, gestational age, infant gender, parity, congenital anomalies, including hypospadias and cryptorchidism, and infant weight.

### Statistical analyses

From 514 participants, ten were excluded from the study due to miscarriage, stillbirth, relocation, or voluntary withdrawal from the study before delivery. There were 493 available maternal blood samples for MEHP measurements. Maternal blood samples collected during hospitalization after delivery were excluded from analysis due to the relatively short biological half-life of DEHP. Two hundreds and ninety-five infant cord blood samples were available for reproductive hormone measurements. Finally, 202 samples were included in the statistical analysis, for which both MEHP levels and reproductive hormone levels had been assessed.

In preliminary data analysis the association between MEHP exposure and the characteristics of mothers and infants were calculated by a Spearman correlation test and a Mann–Whitney U test. Associations between maternal MEHP exposure and infant reproductive hormone levels were first calculated with a Spearman correlation test, and then multivariable linear regression analysis was performed. MEHP levels and the concentration of reproductive hormones were converted to a log10 scale as their data did not fall into a normal distribution. To evaluate whether the relationship between hormone levels and MEHP exposure differs based on sex, a multivariable linear regression model for all study participants was also constructed with the interaction term for hormone levels to sex and MEHP interaction. To improve interpretability, the interquartile range (IQR) for the MEHP concentration and the least squares means (LSM) of log-transformed hormone levels were calculated and back transformed. Linear trends of LSM were tested by modeling IQR as a continuous variable. The first quartile was also compared to the 2nd, 3rd and 4th quartile MEHP, and the P values were adjusted using Bonferroni's correction. The limit of detection (LOD) was determined and half LOD values were used when levels were below the LOD for individual hormones. Inclusion of covariates was based on biological considerations and included: maternal age (continuous), maternal smoking during pregnancy (yes or no), maternal alcohol consumption during pregnancy (yes or no), gestational age (continuous), and the blood sampling week of gestation (continuous). All statistical analyses were performed using JMP pro 10 (SAS Institute Inc., NC, USA).

### Ethical approval

The study was approved by the institutional ethical board for epidemiological studies at Hokkaido University Graduate School of Medicine, Hokkaido University Center for Environmental and Health Sciences, and Nagoya University Graduate School of Medicine, in accordance of with principles of the Declaration of Helsinki. All participants provided written informed consent.

## Results

The characteristics of the participants included in the present study with the corresponding median MEHP concentrations (n = 202) are shown Table 1. In the present study, there were no cases of cryptorchidism or hypospadias included, and all infants were born vaginally. MEHP was detected in 100% of samples and the median concentration was 10.4 ng/mL (IQR: 5.88–15.3 ng/mL). The concentration of MEHP was not significantly associated with any of the maternal or infant characteristics.

**Table 1:** Maternal mono(2-ethylhexyl) phthalate (MEHP) concentrations in relation to the characteristics of mothers and infants.

Characteristics		*n* (%)	Mean ± SD	MEHP (ng/mL)	
				Med. (25^th^–75^th^)	*p*-value
Maternal characteristics					
Age at delivery (years)		202	29.8±4.9	Spearman's ρ = 0.035	0.624^a^
Pre-pregnancy BMI (kg/m^2^)		202	21.1±3.1	Spearman's ρ = 0.002	0.978^a^
Parity	Primiparous	110 (54.5)		10.4 (5.65–15.3)	0.672^b^
	Multiparous	92 (45.5)		10.4 (6.08–15.5)	
Annual household income (million yen per year)	<5	142 (71.0)		10.1 (5.56–15.2)	0.177^b^
	≥5	58 (29.0)		11.7 (6.40–15.7)	
Educational level (years)	≦12	91 (45.0)		10.4 (5.94–14.4)	0.960^b^
	≧13	111 (55.0)		10.5 (5.68–15.5)	
Smoking during pregnancy	Nonsmoker	158 (78.2)		10.5 (6.01–15.6)	0.158^b^
	Smoker	44 (21.8)		7.80 (4.99–14.4)	
Alcohol consumption during pregnancy	Nondrinker	132 (65.3)		10.5 (6.08–16.2)	0.386^b^
	Drinker	70 (34.7)		10.2 (5.34–14.7)	
Type of delivery	Vaginal	202 (100)			
	Caesarian section	0 (0.0)			
MEHP (ng/mL)		202		10.4 (5.88–15.3)	-
Infant characteristics					
Sex	Male	93 (46.0)		10.2 (6.30–14.3)	0.734^b^
	Female	109 (54.0)		10.4 (5.60–16.3)	
Birth weight (g)		202	3138.6±331.3	Spearman's ρ = −0.023	0.376^a^
Gestational age (weeks)		202	39.5±1.0	Spearman's ρ = 0.002	0.959^a^

a *p*-values were calculated by the Spearman's *ρ* test,

b *p*-values were calculated by the Mann–Whitney U test.

Table 2 shows the levels of reproductive hormones among male and female infants. For females, the detected percentage of LH, FSH, and inhibin B was 1%, 0%, and 24.1%, respectively, and thus, these hormones were excluded from further analysis. In addition, INSL3 was measured in only 20 samples from female infants and was consequently also omitted from further analysis.

**Table 2:** Distribution of reproductive hormone concentrations.

	All participants				Males				Females			
	n	Med	(25th–75th)	>LOD (%)	n	Med	(25th–75th)	>LOD (%)	n	Med	(25th–75th)	>LOD (%)
T (pg/mL)	202	85.2	(59.7–113.9)	100	93	97.7	(77.3–124.4)	100	109	70.3	(51.8–102.7)	100
E2 (ng/mL)	202	5	(3.56–7.28)	100	93	5.37	(3.62–6.63)	100	109	4.75	(3.39–6.69)	100
T/E2	202	16.7	(12.2–22.3)	n.d.	93	17.7	(12.9–23.2)	n.d.	109	15.5	(12.0–20.9)	n.d.
P4 (ng/mL)	202	227.3	(182.9–278.8)	100	93	233.8	(186.4–302.6)	100	109	216.9	(175.0–271.8)	100
LH (mIU/mL)	198	<LOD	(<LOD-<LOD)	15.7	91	<LOD	(<LOD-0.81)	33	107	<LOD	(<LOD-<LOD)	1
LH/T		n.d		n.d.	91	0.003	(0.002–0.011)	n.d.	107	n.d.		
FSH (mIU/mL)	197	<LOD	(<LOD-<LOD)	20.3	91	<LOD	(<LOD-0.65)	44	106	<LOD	(<LOD-<LOD)	0
SHBG (nmol/L)	202	15.8	(13.5–18.7)	100	93	16.4	(13.6–19.3)	100	109	15.5	(13.2–18.3)	99.4
T/SHBG	202	5.18	(3.71–7.52)	n.d.	93	5.77	(3.94–8.10)	n.d.	109	4.51	(3.49–6.67)	n.d.
PRL (ng/mL)	199	85.8	(62.0–116.0)	99.5	91	82.9	(66.4–116)	100	109	86.9	(56.7–118.3)	99.1
Inhibin B (pg/mL)	202	22.2	(<LOD-44.0)	58.9	93	43.6	(35.4–58.3)	100	109	<LOD	(<LOD-<LOD)	23.9
INSL3	110	0.27	(0.23–0.31)	100	91	0.28	(0.24–0.32)	100	20	0.18	(0.17–0.23)	100

E2, estradiol; FSH, follicle stimulating hormone; INSL3, insulin like factor 3; LH, luteinizing hormone; LOD, limit of detection; n.d., not determined; P4, progesterone; PRL, prolactin; SHBG, steroid hormone binding globulin; T, testosterone.

The correlations between the MEHP levels and the concentrations of reproductive hormones are shown in Table 3. There were significant negative correlations between the MEHP level and P4, PRL, and inhibin B concentrations for all participants, P4, inhibin B, and INSL3 concentrations among male infants, and PRL concentrations among female infants. The results of our linear regression are shown in Table 4. MEHP level was inversely associated with T/E2 ratio, P4, and inhibin B concentrations in the linear regression model fit to all study participants, and the relationship between MEHP levels and these hormones was not statistically significant between males and females (*P*_internaction_>0.05). In the stratified analyses, inverse associations were statistically significant between MEHP level and T/E2 ratio, P4, inhibin B, and INSL3 concentrations among males, but not females.

**Table 3:** Correlations between MEHP concentrations and hormone levels.

	All participants		Males		females	
	ρ	p-value	ρ	p-value	n	p-value
T (pg/mL)	−0.091	0.198	−0.089	0.398	−0.107	0.269
E2 (ng/mL)	0.015	0.830	0.101	0.334	−0.035	0.716
T/E2	−0.086	0.224	−0.147	0.160	−0.043	0.660
P4 (ng/mL)	**−0.202**	**0.004**	**−0.218**	**0.036**	−0.184	0.056
LH (mIU/mL)	n.d.		−0.024	0.822	n.d.	
LH/T	n.d.		0.075	0.478	n.d.	
FSH (mIU/mL)	n.d.		0.205	0.052	n.d.	
SHBG (nmol/L)	−0.047	0.508	−0.037	0.722	−0.050	0.606
T/SHBG	−0.055	0.436	−0.045	0.668	−0.070	0.470
PRL (ng/mL)	**−0.229**	**0.001**	−0.119	0.260	**−0.301**	**0.002**
Inhibin B (pg/mL)	**−0.235**	**0.001**	**−0.474**	**<0.001**	n.d.	
INSL3 (ng/mL)	n.d.		**−0.241**	**0.022**	n.d.	

E2, estradiol; FSH, follicle stimulating hormone; INSL3, insulin like factor 3; LH, luteinizing hormone; MEHP, mono(2-ehylhexyl) phthalate; n.d., not determined; P4, progesterone; PRL, prolactin; SHBG, steroid hormone binding globulin; T, testosterone.

**Table 4:** Adjusted linear regression coefficients (β) of reproductive hormone levels in cord blood in relation to MEHP.

	All participants (n = 202)					Males (n = 93)				Females (n = 109)			
	β	(95%CI)		p-value^a^	p for interaction^b^	β	(95%CI)		p-value^c^	β	(95%CI)		p-value^c^
T (pg/mL)	−0.147	−0.301	0.006	0.059	0.799	−0.130	−0.352	0.093	0.250	−0.133	−0.339	0.073	0.204
E2 (ng/mL)	0.024	−0.107	0.155	0.718	0.703	0.065	−0.151	0.282	0.549	0.001	−0.160	0.161	0.993
T/E2	**−0.171**	**−0.295**	**−0.048**	**0.007**	0.473	**−0.195**	**−0.365**	**−0.025**	**0.025**	−0.134	−0.306	0.038	0.126
P4 (ng/mL)	**−0.237**	**−0.401**	**−0.074**	**0.005**	0.771	**−0.311**	**−0.528**	**−0.095**	**0.005**	−0.200	−0.430	0.029	0.086
LH (mIU/mL)	n.d.					−0.088	−0.353	0.177	0.510	n.d.			
LH/T	n.d.					0.054	−0.324	0.432	0.777	n.d.			
FSH (mIU/mL)	n.d.					0.171	−0.020	0.361	0.078	n.d.			
SHBG (nmol/L)	0.013	−0.056	0.082	0.709	0.525	0.026	−0.058	0.109	0.542	−0.006	−0.106	0.093	0.900
T/SHBG	−0.160	−0.336	0.015	0.073	0.637	−0.156	−0.384	0.073	0.179	−0.127	−0.376	0.123	0.317
PRL (ng/mL)	−0.080	−0.201	0.041	0.194	0.290	−0.041	−0.181	0.099	0.563	−0.132	−0.312	0.048	0.150
Inhibin B (pg/mL)	**−0.288**	**−0.405**	**−0.170**	**<0.001**	0.970	**−0.276**	**−0.404**	**−0.148**	**<0.001**	n.d.			
INSL3 (ng/mL)	n.d.					**−0.156**	**−0.258**	**−0.054**	**0.003**	n.d.			

Reproductive hormones levels and MEHP concentration were log10-transformed and included in the model separately.

a, adjusted for maternal age, smoking during pregnancy, alcohol consumption during pregnancy, gestational age, blood sampling week, infant sex, and interaction of sex and MEHP.

b, P for interaction of sex and MEHP.

c, adjusted for maternal age, smoking during pregnancy, alcohol consumption during pregnancy, gestational age, blood sampling week.

E2, estradiol; FSH, follicle stimulating hormone; INSL3, insulin like factor 3; LH, luteinizing hormone; MEHP, mono(2-ehylhexyl) phthalate; n.d., not determined; P4, progesterone; PRL, prolactin; SHBG, steroid hormone binding globulin; T, testosterone.

**Figure 1:**
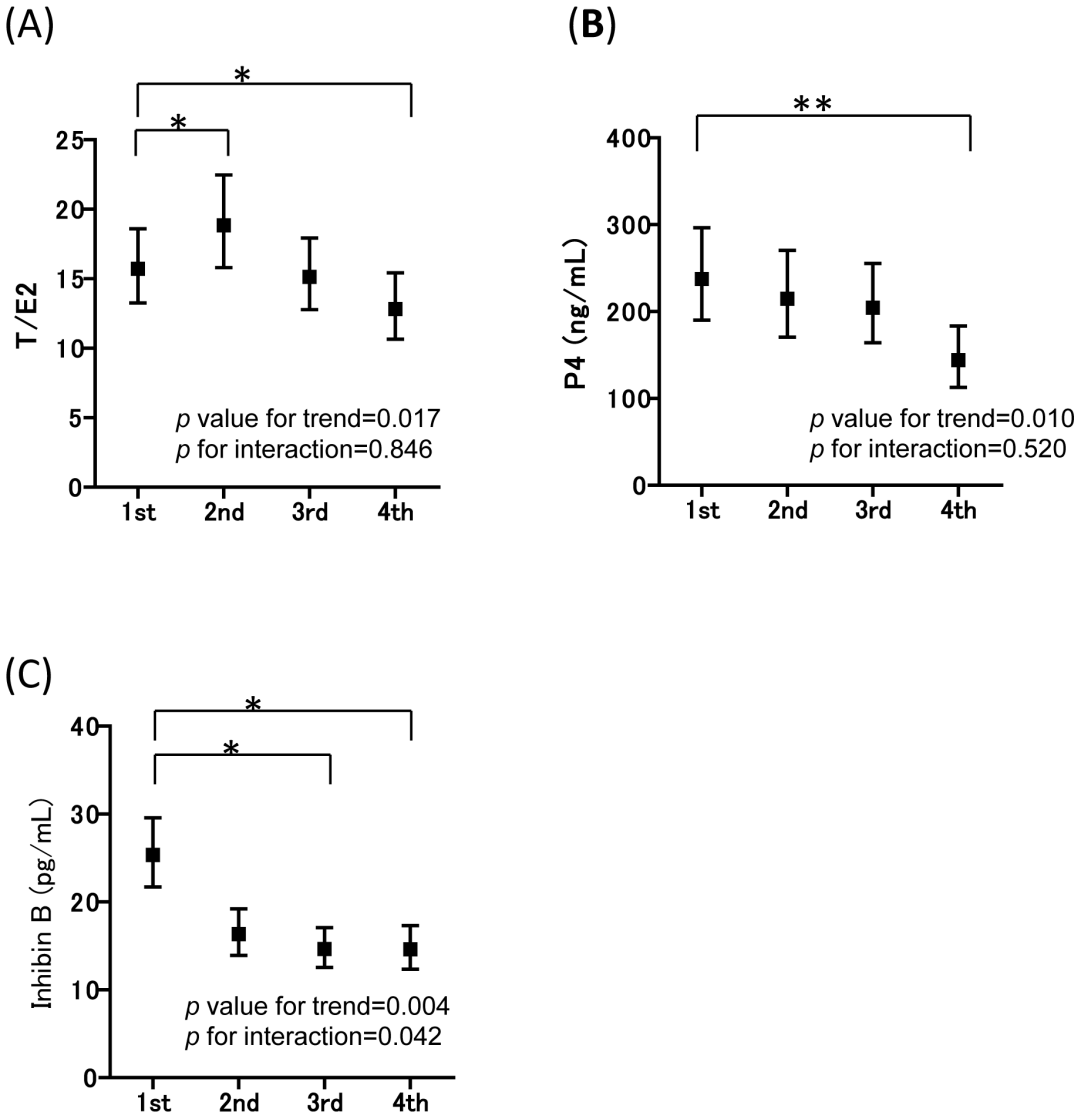
X-axis shows the MEHP quartiles, and Y-axis shows each hormone level. The adjusted LSMs (95% confident intervals) of each hormone level in cord blood in relation to the MEHP concentration quartile fit to all study participants are shown with *p*-value for trend, and *p* for interaction, respectively, for (**A**) T/E2 (0.017, 0.846), (**B**) P4 (0.010, 0.520), and (**C**) inhibin B (0.004, 0.042). First quartile (≦5.90 ng/mL) is also compared to the 2nd (5.91–10.39 ng/mL), 3rd (10.40–15.30 ng/mL) and 4th (15.31+ ng/mL) quartile MEHP. Statistical significance of the P value was *p<0.017, **p<0.002 based on Bonferroni's correction. When compared to the LSM of the 1st MEHP quartile, the 4th MEHP quartile of T/E2, P4, and the 3rd and 4th inhibin B significantly decreased, whereas the 2nd MEHP quartile of T/E2 significantly increased. LSMs were adjusted for maternal age, smoking during pregnancy, alcohol consumption during pregnancy, gestational age, and the blood sampling week, infant sex, and interaction of sex and MEHP.

**Figure 2:**
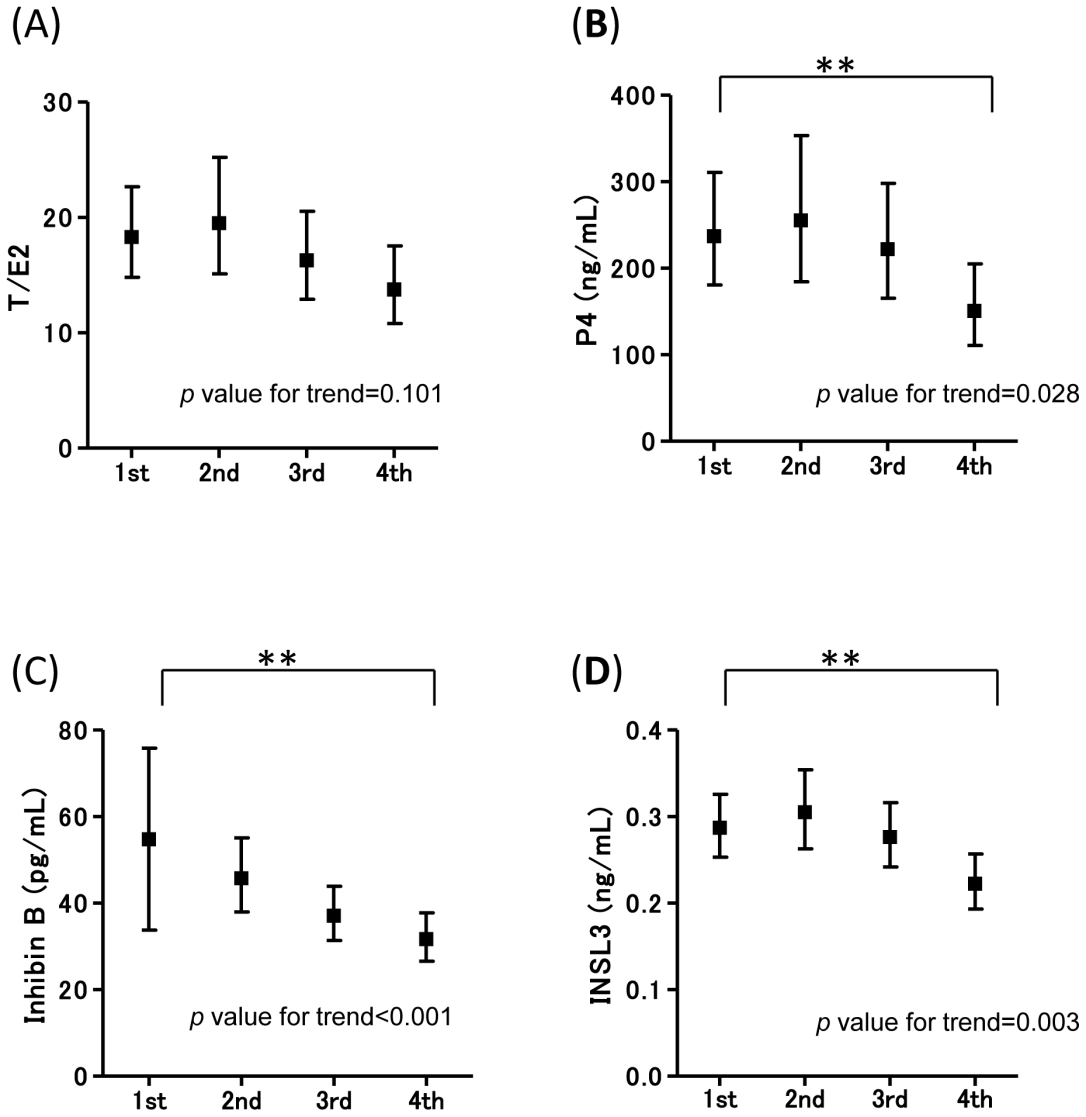
X-axis shows the MEHP quartiles, and Y-axis shows each hormone level. In males, the adjusted LSMs (95% confident intervals) of each hormone levels in cord blood in relation to the MEHP concentration quartile (*p*-value for trend) are (**A**) T/E2 (0.357), (**B**) P4 (0.028), (**C**) inhibin B (<0.001), (**D**) INSL3 (0.005). First quartile (≦6.36 ng/mL) is also compared to the 2nd (6.37–10.25 ng/mL), 3rd (10.25–14.28 ng/mL) and 4th (14.29+ ng/mL) quartile MEHP. Statistical significance of the P value was *p<0.017, **p<0.002 based on Bonferroni's correction. LSMs were adjusted for maternal age, smoking during pregnancy, alcohol consumption during pregnancy, gestational age, and the blood sampling week.

The associations between MEHP and the levels of reproductive hormones in infants were assessed for potential non-linear relationships. The MEHP concentration was divided into four sections and the LSM of each hormone in each MEHP quartile is shown in Figure 1 and Figure 2 for all participants and male infants, respectively. The adjusted LSM hormone levels in relation to the MEHP quartile showed a significant *p*-value trend for T/E2 and P4 in the model fit to all study participants. Sex did not modified the association of either MEHP and T/E2 or P4 (*P*_internaction_>0.05). When compared to the LSM of the 1st MEHP quartile, the 4th MEHP quartile of T/E2, P4 significantly decreased, whereas the 2nd quartile of T/E2 significantly increased. The 3rd and 4th MEHP quartile of inhibin B significantly decreased when compared to the LSM of the 1st quartile, and inhibin B levels differ in the 2nd quartile in the interaction term for MEHP and sex (*P*_interaction_ = 0.008). For sex stratification, the adjusted LSM hormone levels in relation to the MEHP quartile showed a significant *p*-value trend for P4, inhibin B, and INSL3 in males. In addition, when compared to the LSM of the 1st MEHP quartile, the 4th MEHP quartile of P4, inhibin B, and INSL3 significantly decreased.

## Discussion

In the present study maternal MEHP levels were found to be associated with reduced levels of T/E2, P4, and inhibin B in an adjusted analysis when the model was fit to all study participants. For the stratified analyses for sex, inverse associations between maternal MEHP levels and T/E2, P4, inhibin B, and INSL3 were statistically significant for males. INLS3 is a major product secreted by Leydig cells. The testosterone produced by Leydig cells is regulated by a negative feedback loop, which is controlled by the hypothalamic–pituitary–gonadal axis, and is chronically influenced by the long-term differentiation status of the cells [32]. On the other hand, INSL3 is constitutively expressed by Leydig cells, and is thus more advantageous over testosterone as a marker of Leydig cell differentiation [32]. Fetal exposure to phthalates and their effect on Leydig cell development has been the subject of several review papers that have examined evidence from experiment *in vitro* cell studies and animal models [33], [34]. The results obtained from the present study are consistent with previous research.

In the multivariable linear regression model fit to all study participants, MEHP level was inversely associated with T/E2 ratio, and there was no statistical significance between males and females. Similar in Taiwan, Lin et al. [22] found that the fT concentration and fT/E2 ratio in cord blood were inversely correlated with two DEHP metabolites among females. In a Danish–Finnish cohort study, Main and co-workers measured phthalate monoesters in breast milk and found an inverse association between the level of monoesters and the concentration of testosterone, as well as positive associations with the levels of SHBG and the LH/fT ratio in males [23]. An inverse association between T/E2 and MEHP was significant in males but not in females suggesting a more pronounced effect in males than in females in this study. Interestingly, an inverted J-shaped curve was observed for T/E2 and MEHP quartile associations. Andrate et al. observed that DEHP exposure showed aromatase inhibition at low doses and stimulation at high doses in animal study [35]. However, the non-monotonic biological effect of DEHP exposure to aromatase activity within the range of MEHP quartiles in this study is questionable. In this study the MEHP level was found to be inversely related to P4 in infants. Previous *in vitro* research shown that MEHP can suppress steroidogenesis and down-regulate P4 production in MA-10 Leydig cell [36]. Reduced progesterone values in combination with normal testosterone values indicate that the steroidogenesis pathway from progesterone to testosterone is not affected in the DEHP exposure. Meanwhile, an association between MEHP concentration and testosterone level showed p values of 0.059 in all participants. Thus, insignificance of reducing testosterone maybe due to low statistical power, and thus, additional studies with larger sample size are needed.

Sertoli cells may also be the targets of reproductive toxicity induced by phthalate exposure *in utero* [37]. The MEHP level in maternal blood samples was inversely related to inhibin B in cord blood in males suggesting the fetal exposure of DEHP affected Sertoli cells in human infants, although in Main et al., the associations between phthalate monoesters and inhibin B were not clear [23]. These findings are in agreement with animal studies that have shown that neonatal exposure to DEHP reduces Sertoli cell numbers and proliferation in rodents [38], [39]. The establishment of appropriate Sertoli cell numbers during development is critical for the production of sperm in adulthood [40]. Further study to evaluate the long-term effects of DEHP exposure *in utero* on testicular function should be considered. In this study, an inverse association between inhibin B and all participants was also observed, with the significant interaction term for MEHP and sex in quartile model. However, this significance may be due to the low detection rate of inhibin B in females. Therefore, more studies are needed to confirm these results.

The levels of MEHP detected in this study were slightly higher when compared to other populations, with the exception of one study carried out in Italy. For example, the median (IQR) MEHP levels in American adults (NHANES 1999–2000), elderly Swedish subjects, and pregnant women in Australia were 5.4 (3.4–8.9) ng/mL, 4.5 (2.0–15.5) ng/mL, and 1.18 (<LOD, 3.10) ng/mL, respectively [41]–[43]. One study performed with pregnant woman in Italy showed a mean MEHP concentration of 0.68±0.85 µg/mL [44], which is almost two orders of magnitude higher than what has been found in other studies, including the present work. However, the exposure levels of DEHP in this cohort are not externally comparative to previous studies due to the difference in measurement methods of each study. The majority of the recent studies assessed phthalate exposure based on urine samples. Unfortunately, urine samples were not available for the purposes of this cohort study.

It should be noted that, in the present study, only MEHP was measured, and this is a limitation to the study. MEHP is the primary metabolite of DEHP and there are several secondary metabolites, such as mono(2-ethyl-5-hydroxyhexyl) phthalate, mono(2-ethyl-5-oxohexyl) phthalate, mono(2-ethyl-5-carboxypentyl) phthalate, and mono(2-carboxymethylhexyl) phthalate [45]–[47]. In urine samples approximately 70% of detected phthalates are found in one of these four oxidized metabolite forms, whereas only 6% are found in the form of MEHP [47]. However, in blood samples, MEHP is detected in more than 80% of samples and the detection rates of the oxidized metabolites is less than 40% [48]–[50]. Therefore, the analysis of MEHP in blood does provide an indication of DEHP exposure. The associations between MEP and infant T and SHBG levels have been previously reported in three-month-old males, where MEP, monobutyl phthalate, and monobenzyl phthalate were found to be inversely related to the AGI [20], [23]. Other phthalates, such as DEP, DBP, and butyl benzyl phthalate should also be considered in future studies.

It is also important to note that there may be potential contamination of blood samples with DEHP from medical devices, which are additional limitations of the present study. However, speed of diester to monoester conversion of DEHP is relatively longer [49] and the diester hydrolysis was not observed after one hour incubation at 37°C [51]. In this study, after blood samples were taken, they were handled at 4°C until storage at −80°C. In addition, all samples were collected at a single hospital so that any variation of medical devices used for withdraw blood would be low. Thus, although there is a possibility of DEHP contamination, the effect of *ex vivo* hydrolysis would have a low impact on the results. In addition, similar associations were found between the recorded levels of reproductive hormones and MEHP through the use of linear regression and quartile analysis, and this approach could be used in the future to minimize variations in MEHP exposure [14], [41], [52]. Consequently, the inverse associations observed between infant reproductive hormones and the concentration of MEHP in maternal blood should be considered relevant. Although it is unlikely to introduce a positive bias by sample contamination, future studies are needed to confirm the results.

Another limitation of this study is that the majority of maternal blood samples were taken during the third trimester of pregnancy. Therefore, the effects of fetal exposure to DEHP during the earlier stages of fetal development have not been directly assessed. In addition, the MEHP level was measured only once. However, several previous reports have found that single measurements can be useful, although there is some discussion regarding this in the literature [14], [53]–[55]. Lastly, there was a selection bias in this study as only participants with available cord blood samples were included in the analysis. Cord blood samples were only taken in births where the infants were delivered vaginally. Compared to the initial cohort population, the infants included in this study had an increased gestational age and heavier birth weight than the infants that were excluded from the study. Thus, the effects of MEHP may be underestimated in this study.

## Conclusions

This study found that maternal DEHP exposure negatively correlates with the levels of P4, inhibin B, and INLS3 and these associations were more pronounced in male infants than in females. These results suggest that both Leydig and Sertoli cell development may be adversely affected by DEHP exposure *in utero* in males. The investigations of other phthalates in comprehensive studies to facilitate full appreciation of the effects of DEHP exposure, as well as its long-term effects on reproductive development are needed.
